# A Rapid and Quantitative Method for Determining Seed Viability Using 2,3,5-Triphenyl Tetrazolium Chloride (TTC): With the Example of Wheat Seed

**DOI:** 10.3390/molecules28196828

**Published:** 2023-09-27

**Authors:** Shuonan Wang, Mengmeng Wu, Sunyaxin Zhong, Jing Sun, Xinyue Mao, Nianwei Qiu, Feng Zhou

**Affiliations:** 1 College of Life Sciences, Qufu Normal University, Qufu 273165, China; 2 Shandong Freshwater Fisheries Research Institute, Jinan 250013, China; 3 School of Food Science, Nanjing Xiaozhuang University, Nanjing 211171, China

**Keywords:** wheat seed, seed viability, germination stage, 2,3,5-triphenyl tetrazolium chloride (TTC), 1,3,5-triphenylformazan (TTF), dimethyl sulfoxide (DMSO), staining, extraction

## Abstract

Current colorimetric methods for quantitative determination of seed viability (SV) with 2,3,5-triphenyl tetrazolium chloride (TTC) have been plagued by issues of being cumbersome and time-consuming during the experimental process, slow in extraction and staining, and exhibiting inconsistent results. In this work, we introduced a new approach that combines TTC-staining with high-temperature extraction using dimethyl sulfoxide (DMSO). The optimization of the germination stage, TTC-staining method, and 1,3,5-triphenylformazan (TTF) extraction method were meticulously carried out as follows: When the majority of wheat seeds had grown the radicle, and the length of radicles was approximately equal to the seed length (24 h-germination), 2 g germinating seeds were placed into a beaker (20 mL) containing 5 mL 10 g·L^−1^ TTC solution. The seeds were stained with TTC in the dark at 25 °C for 1 h. Following the staining, 1 mL 1 mol·L^−1^ H_2_SO_4_ was added to stop the reaction for 5 min. The H_2_SO_4_ solution was then removed, and the seeds were gently rinsed with deionized water. Subsequently, the TTF produced in the seeds was extracted directly with 5 mL DMSO solution at 55 °C for 1 h. The absorbance of the extract was measured at 483 nm, and the index of SV was calculated according to a predetermined TTC calibration curve and expressed by mg TTC·g^−1^ (seed)·h^−1^. The new method has been demonstrated to be rapid, stable, and highly sensitive, as evidenced by the accurate measurement of seed viability with different aging degrees.

## 1. Introduction

High viability of seeds is crucial for ensuring a productive agricultural yield. Thus, measuring seed viability (SV) has become essential during various processes of breeding, sowing, seed storage, and seed trade. A number of methods have been employed to determine SV, including international rules for seed germination testing [1], the electrical conductivity (EC) method [2,3,4], and the staining method [5,6,7,8,9,10,11]. The germination test method is impractical for assessing multitudinous seed samples due to its time-consuming and labor-intensive nature. The EC method suffers from larger errors influenced by multiple factors [5]. In addition, some seeds require careful breaking of the semipermeable layer for the measurement of EC, which limits the application of the EC method [3]. The staining method with 2,3,5-triphenyl tetrazolium chloride (TTC) has long been the preferred choice for rapid determination of SV [5,6,7,8,9,10,11]. In the TTC method, the dehydrogenase activity in seeds is used to quantify SV. Dehydrogenase can reduce the colorless TTC (soluble in water) to red 1,3,5-triphenylformazan (TTF) (insoluble in water). SV is expressed by the staining intensity of the seed embryo [6,7].

The TTC methods for determining SV can be divided into qualitative determination and quantitative determination. The qualitative determination method with TTC involves cutting open the seed embryo and staining the embryo cells with TTC solution, allowing for quick qualitative assessment of seed viability based on whether the embryo is stained red or not. The percentage of living seeds among the total tested seeds is commonly used to express the quality of seeds [5,12,13]. This method has the advantages of simple operation, quick reaction, and low cost, which is a universal method for quick evaluation of seed quality [1]. However, this method can solely differentiate living seeds from dead seeds and does not offer the capability to quantify seed viability [2,14,15]. At present, an all-in-one scanner has been used to scan and calculate the staining area and intensity of embryos, enabling quantification of SV [16]. However, this scanning method requires cutting open the embryos and is prone to operational errors, making it an immature technology at present.

Living seeds may not always result in robust sprouting seedlings. The vitality of seeds is intricately linked to the strength of the ensuing seedlings. To accurately assess seed quality, quantitative measurement of SV is imperative. However, the current quantitative determination method with TTC is complicated and time-consuming. It involves homogenizing the germinating seeds by grinding, followed by staining with 15 mL 1% TTC. After intricate processes such as centrifugation, drying, and extraction, the red TTF extract is obtained. The absorbance of the TTF extract is measured at 485 nm, based on which SV is quantified. The total experimental process can take up to 2 d [17]. A common hindrance to the TTC-staining method is the presence of a semipermeable layer in the seed coat or the aleurone layer [18,19,20], which prevents TTC from entering the embryo cells. Before TTC-staining, it is necessary to destroy the semipermeable layer by grinding the seeds or stripping the seed coat [10,17,21].

In order to achieve quantitative determination of SV, various new determination methods have been proposed in recent years. Some researchers have assessed SV by the number of radicles or the elongation rate of radicles in germinating seeds in some research [22]. However, this method still suffers from challenges such as large statistical workload, large experimental errors, and prolonged experimental time. Another new method was developed to quantify SV based on the respiratory oxygen consumption rate of germinating seeds [23]. However, because of the low respiration rate of seeds, measuring the respiration rate of seeds requires a large number of seeds and expensive high-precision equipment. Moreover, only one sample can be determined at a time, so it is not suitable for SV determination of multiple seed samples. Other experimental techniques, such as vibrational spectroscopy [24,25] and near infrared reflectance spectroscopy [26], have been proposed but are all still in the trial phase. In this context, the improvement of the TTC-staining method remains a critical pathway towards achieving accurate quantification of SV. A new method for rapid and quantitative determination of SV was proposed in this paper. The germinating seeds were stained with TTC solution directly, and then the formed TTF was extracted quickly with DMSO at high temperature. Seed viability is quantified according to the absorbance of the TTF extract. This new method greatly simplifies the experimental process, shortens the experimental time, and enhances the overall efficiency, representing a promising advancement in seed viability assessment.

## 2. Results and Discussion

### 2.1. The Optimal Germination Stage for the Determination of SV

**Figure 1:**
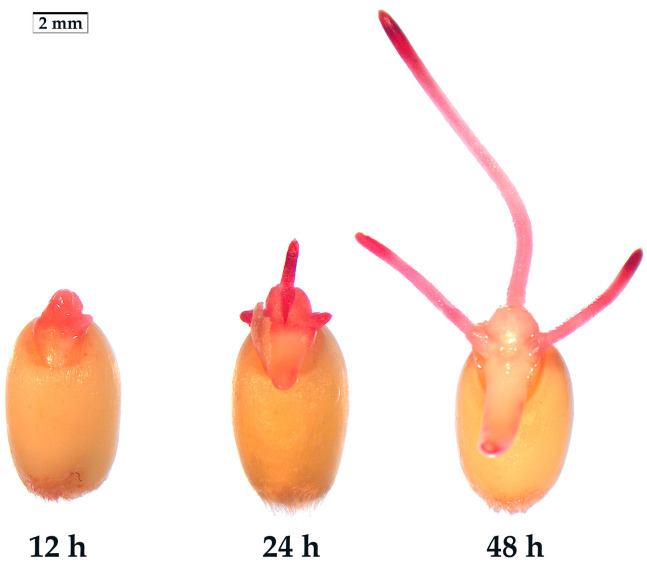
Embryo status after TTC-staining for 1 h. The wheat seeds germinated for 12 h, 24 h, and 48 h at 25 °C, respectively.

The presence of a semipermeable layer in seeds poses a challenge for traditional TTC-staining as it prevents the penetration of TTC into the embryos [18,19,20]. Only when the radicles break through the seed coat does TTC effectively stain the surface cells of the radicles and germs. In order to screen the optimal germination stage for determining SV, the wheat seeds were germinated in an incubator at room temperature (25 °C) for 12 h, 24 h and 48 h, respectively. Subsequently, the seeds were stained with 10 g·L^−1^ TTC solution at room temperature (25 °C) in the dark for 1 h. The staining results showed that the deepest stained part was the radicle. As such, controlling the germination length of the radicle is the key step to measure SV (Figure 1). After germination for 12 h, the radicle of some seeds did not break through the seed coat and could not be stained; the radicle of the other seeds just broke through the seed coat, but the radicle displayed shallow staining. After germination for 24 h, the length of the radicles ranged from 1 to 5 mm, showing deeper radicle staining and relatively lighter germ staining. The 24-h germinating seeds yielded sufficient TTF for SV determination. After a 48-h germination, the radicle length of most seeds was about 5–10 mm. The root tip was deeply stained, and the other parts were stained relatively lightly. At this stage, the determination primarily reflected the viability of radicles rather than the seed embryos. Based on these observations, it is recommended to select the 24-h germination stage, when most seeds have just broken through the seed coat, and the radicle length does not exceed the seed length, as the optimal time for determining SV. However, as different plant seeds exhibit varying germination speeds under different conditions [5], the optimal germination stage for determining SV needs to be decided by pre-experimentation. In comparison to conventional methods that involve cutting open seeds or stripping seed coat for TTC-staining [10,17,21], this new direct TTC-staining method has proven to be significantly less labor-intensive and more efficient.

### 2.2. Screening of TTF Extraction Methods

#### 2.2.1. Screening of Extraction Solvent

Since TTF is insoluble in water but soluble in organic solvents, the extraction of TTF generated within the embryo cells requires the use of organic solvents such as ethyl acetate [27], ethanol [28,29,30], methanol [31], and acetone [32]. In order to investigate and compare the extraction efficiency of these organic solvents, the 24 h-germinating seeds (2 g per sample) were stained with 5 mL 10 g·L^−1^ TTC at 25 °C for 1 h, and the staining reaction was then terminated with 1 mL 1 mol/L H_2_SO_4_ for 5 min [27]. The stained seeds were rinsed with deionized water. After draining the solution, the TTF in the embryos was extracted with 5 mL ethyl acetate, methanol, ethanol, and acetone, respectively, at 25 °C for 12 h. In another extraction method, 24 h-germinating wheat seeds (2 g per sample) were stained, and then the stained embryos were isolated. The TTF in the stained embryos was extracted by grinding with the same four solvents. The extraction efficiency of TTF was quantified by measuring the absorbance (OD_485_) of the supernatant after the extract was centrifuged at 5000× *g* and 25 °C for 10 min.

**Figure 2:**
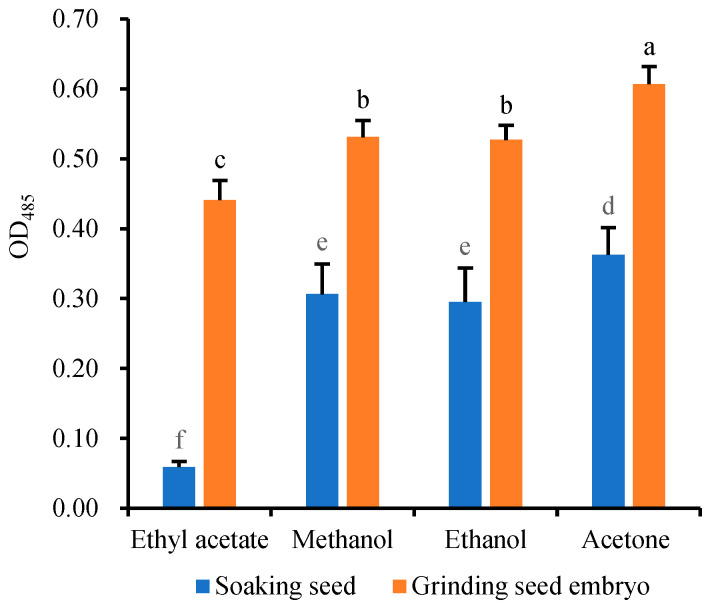
Extraction efficiency of TTF by directly soaking seeds in four organic solvents or soaking in these four organic solvents after grinding the embryos. Means marked with different lowercase letters indicate significant differences (*p* < 0.05).

The results showed that the TTF could not be completely dissolved by any of the four solvents even after 12 h of soaking (Figure 2), and the embryos remained red after the extraction. Ethyl acetate had the lowest extraction efficiency due to its insolubility in water. The direct extraction efficiency of TTF from the stained seeds was ranked as follows: acetone > methanol ≈ ethanol > ethyl acetate. TTF extraction based on these solvents proved to be time-consuming and required heating [29]. However, these four solvents are volatile and cannot be used to extract TTF at high temperatures. Additionally, the tough seed coat made it challenging to grind the stained seeds (2 g) into powder, necessitating the isolation of stained embryos for grinding and TTF extraction using the aforementioned solvents [10,17,21]. The results in Figure 2 indicated that the effect of grinding extraction (OD_485_) from the isolated embryos was significantly better than that of direct extraction from the stained seeds. However, both isolation and grinding of embryos were labor-intensive, and a small amount of red TTF still remained in the grinding residue. As a result, the extraction efficiency of TTF continues to be a bottleneck, restricting the widespread application of the TTC-quantitative method.

#### 2.2.2. Extraction Efficiency of TTF with Dimethyl Sulfoxide (DMSO) at Different Temperatures

DMSO has the advantages of high polarity, high boiling point (189 °C), good thermal-stability, non-volatilization at high temperature, and strong permeability, which can be miscible with acetone, ethanol, chloroform, and water. These attributes render DMSO as a “universal solvent” widely used for rapid extraction of substances like chlorophyll [33].

To validate the extraction efficiency of DMSO, the stained seeds (2 g per sample) were drained of the water and directly soaked in 5 mL DMSO to extract TTF at 25 °C, 40 °C, 50 °C, 60 °C, 70 °C, 80 °C, and 90 °C, respectively. The time required for complete extraction of TTF (embryos whitening) and the absorbance (OD_485_) of the extract are recorded in Table 1. The results showed that with the increase of extraction temperature, the extraction time gradually shortened. Remarkably, at 60 °C, it only took approximately 60 min to achieve embryo whitening. Nevertheless, the extract became turbid due to the gelatinization of starch in wheat seeds. The gelatinization temperature of wheat starch typically begins around 60 °C, peaks at approximately 65 °C, and concludes around 85 °C [34,35]. Below 60 °C, the DMSO extract of TTF remained clear and transparent, rendering it suitable for direct absorbance determination of TTF. Therefore, a temperature of 55 °C is recommended for TTF extraction in starchy seeds. The results in Table 1 also demonstrated the stability and solubility of TTF in DMSO at temperatures ≤ 60 °C, as evidenced by the consistent absorbance readings between 25 °C and 60 °C.

### 2.3. Absorption Spectra of TTF in DMSO and Calibration Curve of TTC

#### 2.3.1. Absorption Spectra of TTF in DMSO

**Figure 3:**
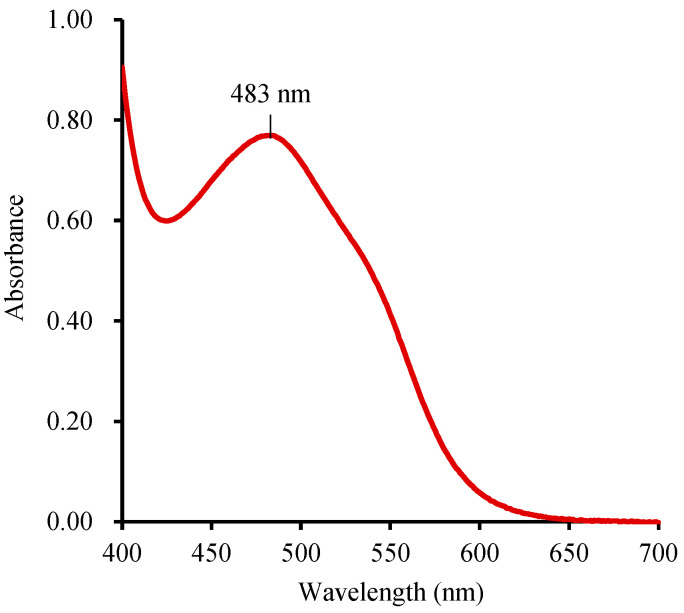
The absorption spectrum of TTF in DMSO.

The wavelength for quantification of TTF varies from 480 nm to 530 nm [21], in which 485 nm is generally used for quantification of the formatted TTF [7,8,21,29,32]. The absorption peaks of the same substance may exhibit slight variations in different solutions. To ensure precise measurements, the absorption spectra of TTF in DMSO were measured by a spectrophotometer. In this paper, Na_2_S_2_O_4_ was employed as a reducing agent to convert TTC to TTF due to its faster reaction rate and cost-effectiveness [7,36]. By scanning the range of 400–700 nm, the maximum absorption wavelength of TTF in DMSO was determined to be 483 nm (Figure 3). While wavelengths between 480 nm and 485 nm showed little difference in the OD values of the TTF solution. It is advisable to use 480–485 nm as the determination wavelength in DMSO. Employing 483 nm can ensure precision in TTF quantification, enhancing the reliability of the quantitative determination of seed viability.

#### 2.3.2. TTC Calibration Curve and Calculation Formula of SV

**Figure 4:**
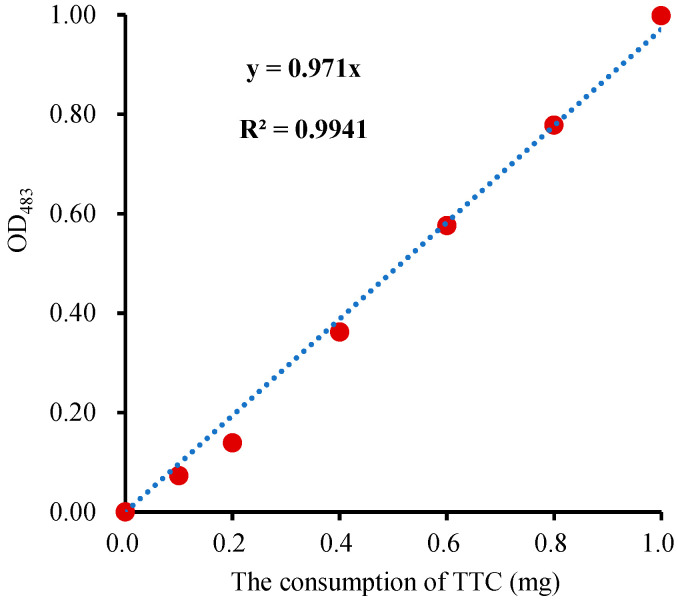
The TTC-OD_483_ calibration curve.

To create a robust calibration curve for seed viability calculation, TTC solutions (1 mL each) with a concentration of 0.2, 0.4, 0.6, 0.8, and 1.0 g·L^−1^ were individually mixed with Na_2_S_2_O_4_ (10 mg powder) to convert TTC to TTF. Subsequently, the reaction solution was evaporated to dryness at 105 °C, and the residue (TTF) was dissolved with 5 mL DMSO for the measurement of OD_483_. As one molecule of TTC can be reduced to one molecule of TTF, the consumption of TTC is proportional to the production of TTF. Therefore, the calibration curve was plotted with the OD_483_ as the ordinate and the consumption of TTC (C_TTC_) as the abscissa (Figure 4). There was a significant correlation between C_TTC_ and OD_483_ (*p* < 0.05) in the calibration curve, and the correlation coefficient (R) was 0.9994, which indicated that the calibration curve was accurate. Alternatively, the calibration curves can also be plotted with the TTF and OD_520_ [28]. However, this approach requires standard TTF, which escalates the overall measurement cost. The reduction consumption of TTC in the sample (C_TTC_) could be obtained according to the regression equation of C_TTC_-OD_483_ (Figure 4). Seed viability (SV) is expressed as the consumption of TTC reduced per gram of germinating seed per hour and calculated by the following formula:

C_TTC_ (mg) = OD_483_/0.971

SV (mg TTC·g^−1^FW·h^−1^) = R_TTC_/FW (g)/time (h)

### 2.4. Screening TTC-Staining Concentration and Time

#### 2.4.1. Screening the TTC-Staining Concentration

**Figure 5:**
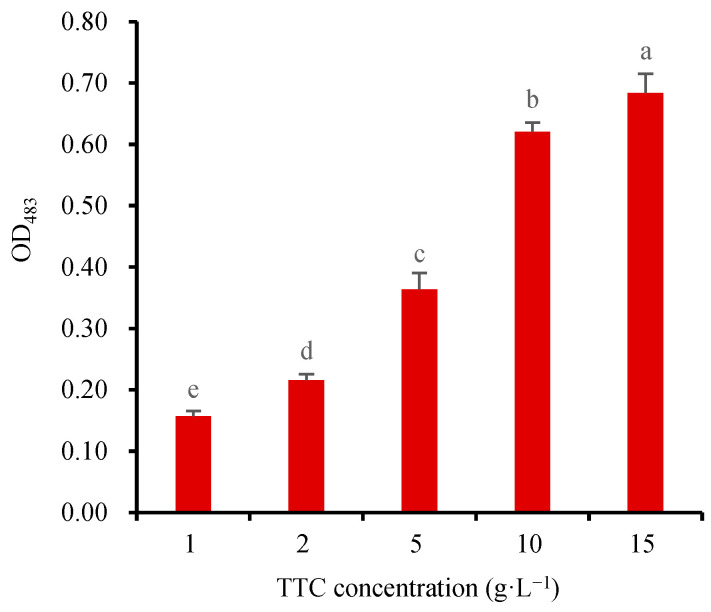
Relationship between the TTC-staining concentration and the absorbance (OD_483_) of the TTF extract (The seeds were stained at 25 °C for 1 h). Means marked with different lowercase letters indicate significant differences (*p* < 0.05).

To determine the optimal TTC-staining concentration for assessing wheat seed viability, 24-h germinating wheat seeds (2 g) were subjected to TTC-staining using varying concentrations of 1, 2, 5, 10, and 15 g·L^−1^ TTC solutions in a 20 mL beaker at 25 °C for 1 h. According to the above method, the TTF generated in the seeds was directly extracted by 5 mL DMSO at 55 °C. The OD_483_ of the extract was measured to screen the optimal TTC-staining concentration. The absorbance results showed that the value of OD_483_ increased gradually with the increase of the TTC concentration (Figure 5). Higher TTC concentrations allowed more TTC to penetrate the embryos, resulting in greater TTF production. A significant OD_483_ increase was observed from 1 g·L^−1^ to 10 g·L^−1^ TTC, while only a smaller increase was observed from 10 g·L^−1^ to 15 g·L^−1^ TTC. Excessive TTC may adversely affect the permeability of the embryo cell membrane, thus affecting its normal respiratory activity. Therefore, the 10 g·L^−1^ TTC solution was selected as the optimal choice for staining wheat seeds, which has also been widely used to measure the SV of other plant seeds [3,11,12,13,15,31,37]. Furthermore, using 10 g·L^−1^ TTC resulted in a clear and transparent TTF extract, and the value of OD_483_ was within a suitable range. Although some studies have reported staining concentrations of 1 or 5 g·L^−1^ TTC, these are relatively uncommon [8,38]. For the seeds with poor permeability of the seed coat, the staining concentration of TTC could be increased to 20 g·L^−1^ [10]. In order to further enhance the permeability of the seed coat, 0.9% (*w*/*v*) NaCl and 2 mmol·L^−1^ DMSO could be added to the TTC solution [10].

#### 2.4.2. Screening the TTC-Staining Time

**Figure 6:**
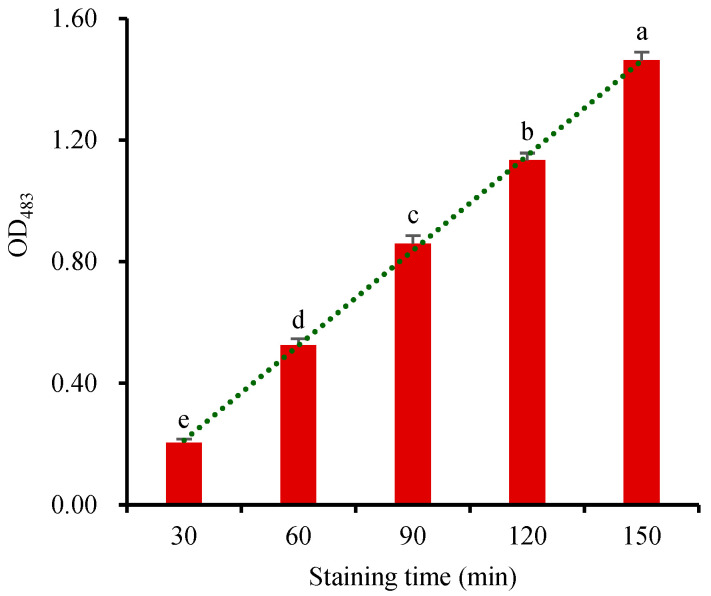
Changes in OD_483_ of the TTF extract of the seeds stained for different durations. Means marked with different lowercase letters indicate significant differences (*p* < 0.05).

The reduction of TTC reflects the respiration intensity of seed embryos. Its reaction rate is related to staining temperature and staining time [28,29,31]. The staining reaction is usually carried out at room temperature (25~30 °C) in the dark [3,11,12,13,15,31,37]. Each wheat seed sample (2 g) was stained with 5 mL 10 g·L^−1^ TTC for 30, 45, 60, 90, or 120 min, respectively. The TTF in the stained seed was directly extracted with 5 mL DMSO at 55 °C. The OD_483_ of the TTF extract showed that the concentration of TTF increased linearly with the extension of the staining time (Figure 6). Therefore, the respiration rate of the seeds remained stable within 150 min, providing a suitable window for stable seed viability measurement. Staining the seeds for 60 min emerged as the optimal duration for accurate OD_483_ determination in this method. However, it is important to note that the viability of different seeds would differ significantly during various germination stages, which may necessitate TTC-staining times ranging from 1 h to 48 h in different studies [3,11,12,13,15,31,37]. During the stage when the radicles sprout out the seeds, the embryos exhibited strong respiratory activity and more easily stained by TTC. Consequently, shorter staining times can be employed at this stage, effectively reducing the staining duration, and enhancing experimental efficiency.

### 2.5. Summary of the Improved TTC-Quantitative Method

The proper amounts of intact and full wheat seeds were sprinkled on a culture dish filled with deionized water. The ventral groove of the wheat seed faced down, and the water depth was maintained at half the height of seeds. The culture dishes were placed in an incubator of 25 °C. The water in the dishes should be replaced every 12 h. Once most of the seeds grew obvious radicles (24 h-germination), the germinating seeds (2 g) were transferred to a 20 mL-beaker containing 5 mL 10 g·L^−1^ TTC solution and stained in the dark at 25 °C for 1 h. At the end of the staining, 1 mL 1 mol·L^−1^ H_2_SO_4_ was added into the beaker immediately to stop the reaction for 5 min. Afterward, the H_2_SO_4_ solution was poured out, and the seeds were gently rinsed with deionized water. After draining the water, the TTF produced in the stained seeds was directly extracted with 5 mL DMSO at 55 °C until the embryos turned white (about 1 h). The absorbance of the extract solution was measured at 483 nm. The index of SV was calculated according to the TTC calibration curve and the value was expressed by “mg TTC·g^−1^ (seed)·h^−1^”. There are some important considerations in this improved TTC-quantitative method, which were briefly described as follows.

(1) TTC is sensitive to strong light and can easily decompose. Attention should be paid to avoiding strong light when preparing the TTC solution and staining the seeds. It is recommended to stain the seed with a fresh TTC solution in the dark at room temperature.
(2) For each seed viability measurement, a new calibration curve should be plotted. It is recommended to use a small beaker as the reaction vessel for the formation reaction of TTF when plotting the calibration curve. The evaporation time could be shortened in a beaker because of its big evaporation area.
(3) Seed viability should be determined when the radicles just sprout out of the seed coat, and the optimal length of the radicle is approximately equal to the seed length.
(4) It is suggested to stain the seeds in a small beaker to ensure sufficient oxygen supply for seed respiration.
(5) If the seed coat is colored and soluble in DMSO, it is necessary to remove the seed coat before TTC-staining to eliminate potential interference.
(6) The staining TTC concentration and staining time should be adjusted by pre-experiments according to seed viability and staining environment.
(7) The TTF extract should be gently mixed and cooled to room temperature before the measurement of OD_483_. If the TTF extract is turbid, the extract should be centrifuged at 5000× *g* for 5 min at room temperature or filtrated before the measurement of OD_483_.
(8) In the staining and extracting processes, make sure both the TTC solution and DMSO completely submerge the seeds.
(9) Maintaining consistent experimental conditions and procedures is necessary for comparing the viability of different seed samples.

### 2.6. Efficiency, Stability, and Sensitivity of the Improved TTC-Quantitative Method

**Figure 7:**
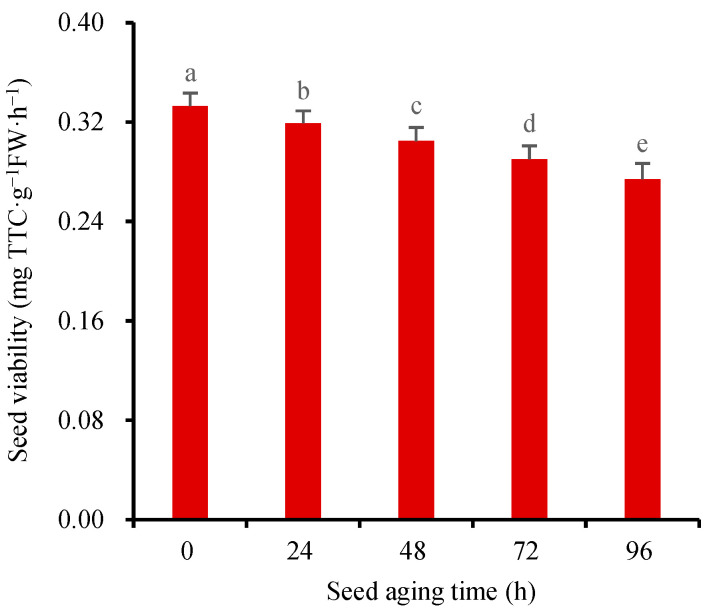
The changes of seed viability at different aging times. Means marked with different lowercase letters indicate significant differences (*p* < 0.05).

The gradual decline in seed viability with the extension of seed storage time, commonly known as “seed aging”, poses an inevitable challenge in seed storage. To simulate the natural aging process of seeds in scientific research, artificial methods are commonly employed to accelerate the aging process. The common methods include hot–humid aging [10,39], methanol aging [40], hot water aging [41], and saturated salt aging [42]. We used the hot–humid method to age the wheat seeds at 40 °C for 0 h, 24 h, 48 h, 72 h, and 96 h, respectively. The improved TTC-quantitative method was used to measure the SV at different aging times. The results showed that the SV of the wheat seeds decreased rapidly with the increase in the aging time (Figure 7). The coefficient variation of SV was less than 10%, which proved that this new method had good stability and reliability. An additional advantage of this approach is that it eliminates the need to strip the seed coat and grind for extraction, making the process simpler and yielding more accurate and reliable experimental results. Furthermore, the improved TTC-quantitative method substantially enhances experimental efficiency, enabling the completion of 25 or more samples in just one morning.

## 3. Materials and Methods

### 3.1. Seeds Materials and Germination Methods

Wheat seeds (*Triticum aestivum* L. Jimai 22) were used as experimental material in this study. The intact and full seeds were sprinkled on a culture dish filled with deionized water. The ventral groove of the wheat seed was faced down and the water depth was half the height of seeds. The culture dishes were placed into a constant temperature incubator (ZGZ-350D, Shanghai Binglin Electronic Technology Co., Ltd., Shanghai, China) of 25 °C, and the water in the dishes was replaced every 12 h. A small number of germinating seeds were taken out every 12 h to screen the optimal germination stage for the determination of SV.

### 3.2. TTC-Staining Method and TTF Extraction Method

The wheat seeds (2 g) that had grown the radicle were stained with 5 mL TTC (purity 98.6%, Sigma Reagent Co., St. Louis, MO, USA) solutions with different concentrations in a 20 mL beaker in a dark incubator of 25 °C to screen the optimal staining concentration of TTC and staining time [43]. During staining, the beaker was gently shaken several times to ensure adequate oxygen supply. At the end of the staining, 1 mL 1 mol/L H_2_SO_4_ was added to each beaker to stop the respiration of seeds [27]. After stopping the reaction for 5 min, the solution in the beaker was discarded. The seeds were rinsed with deionized water once. After draining the water, 5 mL DMSO or other organic solvents were added to the beaker to extract the TTF in the stained seeds. The beakers were placed in the incubators with different temperatures until all TTF was extracted completely. According to the above procedure, the optimal staining concentration of TTC and staining time could be obtained. The optimal extraction temperature and extraction time of TTF could also be obtained by the above procedure. Other conventional reagents used in this research were all analytically pure (≥99.7%) and purchased from Nanjing Chemical Reagent Co., Ltd. (Nanjing, China).

### 3.3. The TTC Calibration Curve and the Calculation of SV

The relationship between the absorbance (OD) of TTF and TTC concentration was determined using a series of standard solutions of 0.2, 0.4, 0.6, 0.8, and 1.0 g·L^−1^ TTC. Each TTC standard solution (1 mL) and 10 mg sodium dithionite (Na_2_S_2_O_4_) powder was mixed in a 10 mL-beaker, respectively, in which TTC was completely reduced by Na_2_S_2_O_4_ to TTF [7,36]. The beakers were then placed into a 105 °C air-blast drying oven and evaporated to dryness [28]. The TTF produced in each beaker was dissolved with 5 mL DMSO. The absorption spectrum (wavelength accuracy 0.1 nm) and the absorbance of the TTF extract were measured by an ultraviolet-visible spectrophotometer (Evolution 350, Thermo Fisher, Waltham, MA, USA). The calibration curve was plotted with the OD values of the TTF extract and the corresponding TTC consumption. According to the calibration curve, the reduction rate of TTC in the germinating seeds was calculated, which was used to quantify the viability of the wheat seeds.

### 3.4. Methods of Seed Aging

Aging of wheat seeds was accelerated under high temperature and high humidity conditions [39]. Dry wheat seeds (20 g) were wrapped in gauze and hung in a 1 L-jar (filled with 400 mL water) above the water level. The covered jar was placed into an incubator at 40 °C and kept for 24 h, 48 h, 72 h, and 96 h, respectively. The viabilities of seeds aged for different durations were quantitatively determined by the new TTC-staining method to validate its stability, sensitivity, and efficiency.

### 3.5. Statistical Method

All data were presented as the mean ± SD. The means were calculated from five replicates of each variant. Significance analysis among the different groups was analyzed by a one-way or two-way ANOVA, followed by Duncan’s multiple comparison. Different superscript letters in the figures were used to denote significant differences (*p* < 0.05)

## 4. Conclusions

Seed viability is affected by its own aging degree and genetic factors [44], as well as environmental factors such as water potential, temperature, and oxygen supply [45]. Rapid and quantitative determination of SV is often needed in agricultural production and scientific research. In this study, wheat seeds were used as experimental material to optimize various parameters, including the germination stage, TTC-staining concentration and staining time, TTF extraction solvent (DMSO), TTF extraction temperature, and accurate TTF absorption wavelength (in DMSO). The plotting method of the TTC calibration curve was also improved. The improved TTC-quantitative method significantly shortens the staining and extraction time and enhances the experimental efficiency. It offers the advantages of simpler procedures and provides accurate and reliable experimental results, making it a valuable method for quantitatively measuring seed viability. This new method will also play an important role in the viability test of a large number of seed samples in genetic breeding and agricultural production.

## Figures and Tables

**Table 1:** Extraction time and extraction efficiency of TTF by DMSO at different temperatures.

Temperature (°C)	Time (min)	OD_485_
25	214 ± 6 ^a^	0.621 ± 0.017 ^a^
40	102 ± 5 ^b^	0.613 ± 0.015 ^a^
50	73 ± 4 ^c^	0.617 ± 0.011 ^a^
60	58 ± 4 ^d^	0.611 ± 0.013 ^a^
70	45 ± 3 ^e^	turbid
80	32 ± 2 ^f^	turbid
90	26 ± 2 ^g^	turbid

Notes: Means marked with different lowercase letters indicate significant differences (*p* < 0.05).

## Data Availability

The data are available from the authors upon request.
